# Substance Use Disorder in Adult-Attention Deficit Hyperactive Disorder Patients: Patterns of Use and Related Clinical Features

**DOI:** 10.3390/ijerph17103509

**Published:** 2020-05-17

**Authors:** Vincenza Spera, Alessandro Pallucchini, Marco Maiello, Marco Carli, Angelo G. I. Maremmani, Giulio Perugi, Icro Maremmani

**Affiliations:** 1 PISA-School of Experimental and Clinical Psychiatry, 56100 Pisa, Italy; e.spera@hotmail.it (V.S.); pallucchini.a@gmail.com (A.P.); marcomaiello@aol.com (M.M.); angelo.maremmani@uslnordovest.toscana.it (A.G.I.M.); 2 School of Clinical Pharmacology, University of Pisa, 56100 Pisa, Italy; c.marco90@hotmail.it; 3 Department of Psychiatry, North-Western Tuscany Region NHS Local Health Unit, Versilia Zone, 55049 Viareggio, Italy; 4 Association for the Application of Neuroscientific Knowledge to Social Aims (AU-CNS), 55045 Pietrasanta, Lucca, Italy; 5 Department of Clinical and Experimental Medicine, Section of Psychiatry, University of Pisa, 56100 Pisa, Italy; giulio.perugi@med.unipi.it; 6 G. De Lisio Institute of Behavioral Sciences, 56100 Pisa, Italy; 7 Vincent P. Dole Dual Disorder Unit, Santa Chiara University Hospital, University of Pisa, 56100 Pisa, Italy

**Keywords:** attention deficit hyperactivity disorder, adult ADHD, substance use disorder

## Abstract

Background: While a large amount of medical literature has explored the association between Attention Deficit/Hyperactivity Disorder (ADHD) and Substance Use Disorders (SUDs), less attention has been dedicated to the typologies of SUD and their relationships with ADHD-specific symptomatology and general psychopathology in dual disorder patients. Methods: We selected 72 patients (aged 18–65) with a concomitant SUD out of 120 adults with ADHD (A-ADHD). Assessment instruments included the Diagnostic Interview for ADHD in adults (DIVA 2.0), Conner’s Adult ADHD Rating Scales–Observer (CAARS-O:S): Short Version, the Structured Clinical Interview for Axis I and II Disorders (SCID-I), the Barratt Impulsiveness Scale (BIS-11), the Brief Psychiatric rating scale (BPRS), the Reactivity Intensity Polarity Stability Questionnaire (RIPoSt-40), the World Health Organization Disability Assessment Schedule (WHODAS 2.0) and the Morningness-Eveningness Questionnaire (MEQ). A factorial analysis was performed to group our patients by clusters in different typologies of substance use and correlations between SUDs, as made evident by their typological and diagnostic features; in addition, specific ADHD symptoms, severity of general psychopathology and patients’ functionality were assessed. Results: Two patterns of substance use were identified: the first (type 1) characterized by stimulants/alcohol and the second (type 2) by the use of cannabinoids (THC). Type 1 users were significantly younger and had more legal problems. The two patterns were similar in terms of ADHD-specific symptomatology and its severity at treatment entry. No differences were found regarding the other scales assessed, except for lower scores at MEQ in type 1 users. Conclusions: At treatment entry, the presence of different comorbid SUD clusters do not affect ADHD-specific symptomatology or severity.

## 1. Introduction

Attention Deficit Hyperactive Disorder (ADHD) and Substance Use Disorder (SUD) are two disorders that are mutually interconnected, as demonstrated by many studies in the literature. About 15% of young adults with ADHD (A-ADHD) have a comorbid SUD [1]. Similarly, a recent meta-analysis estimated the prevalence of ADHD among SUD patients as being about 23.1% [2]. Generally, SUD is more severe in patients affected by ADHD; indeed, the comorbidity between SUD and ADHD is associated with an earlier age at onset of substance use, a higher likelihood of use of a variety of substances and higher rates of polysubstance use [3]. Other studies have found that ADHD in subjects with SUDs is associated with a higher likelihood of suicide attempts, more hospitalizations, a lower likelihood of achieving abstinence, and less treatment compliance [4,5].

Several hypotheses have been proposed to explain the strong association between SUD and ADHD. Familial studies confirmed a common genetic matrix between ADHD and SUD; indeed, the prevalence of SUD seems higher in the parents of ADHD children and in the first-degree relatives of ADHD subjects compared with healthy controls. Similarly, ADHD prevalence is higher in children of parents with SUD. All of these data suggest a common familial and genetic substrate for ADHD and SUD [6,7,8]. Known risk factors for SUD such as impulsivity traits and “sensation seeking” behavior are features shared with ADHD. So too, executive dysfunction and poor judgment may lead ADHD subjects to try substances, so increasing their vulnerability to addiction when compared with their non-ADHD peers [9]. ADHD individuals frequently report difficulties in delaying or modulating reward response, showing a strong tendency to develop addictive behaviors. In this connection, ADHD and SUDs individuals tend to prefer “smaller sooner” over “larger later” rewards [10]. In other words, one major finding is that patients with ADHD prove to be prone to impairment of self-control, and to lack the ability to choose an action ‘now’ to avoid a ‘later’ consequence [11]. The increased exposure of ADHD subjects to psychosocial risk factors, including academic issues and underachievement, interpersonal difficulties and multiple context failures could result in an earlier exposure to addictive drugs deriving from attempts to cope with these deficits [12].

Furthermore, many patients report the use of drugs as a way to suppress ADHD symptoms, following the self-medication hypothesis [13]; according to the latter, people may use addictive drugs as a form of self-therapy in order to relieve painful psychological states through a drug preference related to a psychopharmacological specificity. In this way, ADHD patients may use stimulants paradoxically, to reduce and combat mental and physical restlessness, inattentiveness and emotional moodiness due to dopaminergic dysregulation [14]. In this regard, dopamine neurotransmission has been implicated both in SUD and ADHD. In ADHD, in particular, the neural circuits involved in impulsivity and reward become deficient because of an alteration in dopaminergic transmission; on the other hand, stimulants and other drugs of abuse are able to increase dopamine levels in the mesolimbic reward system (especially in the nucleus accumbens)—a development that may alleviate symptoms of inner restlessness and inattention. The substances mostly used by these patients are alcohol and/or nicotine, cannabinoids, stimulants (amphetamines and cocaine) and opiates. According to a developmental perspective, ADHD symptoms usually appear before adolescence, which suggests a role of ADHD as a risk factor for SUD and not the reverse. Longitudinal studies conducted on non-ADHD children have found several features and behaviors belonging to the traits of “behavioral under-control” and “disinhibition” that, when present during childhood, predict a later diagnosis of ADHD and SUD. Likewise, a large number of prospective studies examined SUD risk trajectories in children with ADHD, showing a heightened risk of psychoactive substance use disorders [15]. For instance, a longitudinal study reported a more frequent marijuana use, daily smoking and alcohol problems in adolescents with a history of childhood ADHD, when compared with a non-ADHD control group [16]. A meta-analysis found that childhood ADHD implies a significant increase in the odds of having used nicotine or illicit drugs, and predicts the likelihood of developing adult nicotine, alcohol, marijuana and cocaine use disorders [15]. The presence of ADHD seems to influence SUD progression by accelerating the transition from “soft” drugs and alcohol to “hard” drug addiction and worsening the prognosis of SUD. All ADHD subtypes are associated with SUD, even if the combined type is more strongly associated with externalizing disorders [17]. It should be noted that attention deficits and symptoms affecting the hyperactivity/impulsivity domain are differently correlated with substance use outcomes [15]. The association between the inattentive subtype and SUD could be explained by the need to reduce the neurocognitive deficits associated with ADHD. Psychic restlessness and hyperactivity lead to substance use as self-medication, in an attempt reduce internal tension and improve emotional self-regulation. Several studies have shown that emotional dysregulation with hyperactive-impulsive symptomatology (HI) may, most likely, be associated with this high risk of substance use [18,19].

In summary, A-ADHD/SUD comorbidity has frequently been reported in the literature but, despite the self-medication hypothesis, these patients are using various substances that have opposite effects. What is more, studies about the influence of these substances on the clinical picture are lacking. Official statements go no further than declaring that comorbid patients have a more severe illness with a worse outcome.

The main purpose of the current study has been to explore substance use patterns among A-ADHD patients while comparing the demographic, clinical and functional features occurring in the different patterns found for substance use. To the best of our knowledge this is the first study ever conducted on a sample of A-ADHD patients never treated with specific ADHD medications that explores patterns of substance use and their clinical correlations at treatment entry.

## 2. Materials and Methods

### 2.1. Design of the Study

This is an observational, cross-sectional, non-interventional study based on a single evaluation of adult patients admitted between 2016 and 2019 to the Outpatient Unit of the Second Psychiatric Unit of the University of Pisa. All the patients recruited in the study were evaluated by clinical interview and specific diagnostic tests by residents of the Department of Clinical and Experimental Medicine, School of Psychiatry, of the University of Pisa, under the supervision of senior psychiatrists of the ADHD research group.

This study was conducted according to the WMA Declaration of Helsinki—Ethical Principles for Medical Research Involving Human Subjects. All subjects examined filled out and signed an informed consent document to qualify for participation in this study. Both the consent form and the experimental procedures were approved by the ethics committee of the University of Pisa (study ID: 14003; code: ADHD-MOOD), in accordance with internationally accepted criteria for ethical research.

### 2.2. Sample

At the treatment entry we consecutively recruited 120 patients with A-ADHD; among these we selected 72 individuals who also met DSM-5 criteria for a Substance Use Disorder. All the recruited patients were diagnosed and treated for the first time after reaching the age of 18.

### 2.3. Instruments

The diagnosis of ADHD was made according to DSM-5 criteria and confirmed using the semi-structured, clinician-administered Diagnostic Interview for ADHD in adults (DIVA 2.0).

Conner’s Adult ADHD Rating Scales-Observer: Short Version (CAARS-O:S) is a 26-item questionnaire completed by a designated person in order to assess DSM-IV Inattentive Symptoms, DSM-IV Hyperactive-Impulsive Symptoms, DSM-IV Total ADHD Symptoms and the ADHD Index; high scores of items in the ADHD Index suggest clinically significant levels of ADHD symptoms. An anamnestic scale was used to assess patients’ demographic and retrospective clinical data. The Structured Clinical Interview for Axis I Disorders (SCID-I) was used for the assessment of any other psychiatric diagnosis, according to DSM-5. For each patient the clinician completed the 18-item version of the Brief Psychiatric Rating Scale (BPRS), a tool commonly used to assess current psychopathology and severity of symptoms. The Hypomania Checklist (HCL-32) questionnaire was used for lifetime history of hypomanic symptoms. The Barratt Impulsiveness Scale (BIS-11) is a 30-item questionnaire used to assess the impulsiveness construct, which is subdivided into three second order domains named Attentional, Motor and Non-Planning Impulsivity. The Reactivity Intensity Polarity Stability Questionnaire (RIPoSt-40) is a self-report questionnaire of 40 items used to measure Emotional Dysregulation (ED) that could be further subdivided into four subscales known as Emotional Impulsivity, Positive Emotionality, Negative Emotionality and Affective Instability. The World Health Organization Disability Assessment Schedule (WHODAS 2.0), developed by the World Health Organization, is a 36-item self-administered questionnaire used to explore functioning and disability in major life domains. The 19-item Morningness-Eveningness Questionnaire (MEQ) was used to assess patients’ chronotype: a total score of 41 or below indicates an “evening chronotype” while a score of 59 or above indicates a “morning chronotype”.

### 2.4. Data Analysis

In order to group our patients by clusters in different typologies of substance use we used the following procedure. We scaled the use of each substance by 4 ordinal levels: 0 = no life-time use; 1 = past use; 2 = current (previous 6 months) use; 3 = past and current use. A factorial analysis was performed on the substances investigated (cannabinoids (THC), cocaine, amphetamines, 3,4-metilenediossimetanfetamina (MDMA), opiates, alcohol, illegal benzodiazepines) in A-ADHD patients in order to identify possible composite dimensions. The initials factors were extracted by means of principal component analysis (PCA-type 2) and then rotated according to Varimax criteria in order to achieve a simple structure. This simplification is equivalent to maximizing the variance of the squared loading in each column. The criterion used to select the number of factors was an eigenvalue >1. Items loading with absolute values >0.40 were used to describe the factors. This procedure makes it possible to minimize the correlations between the factors, so allowing their optimization as classificatory tools for each subject. The factor scores were then standardized as z-scores to facilitate the comparison of scores occurring among the factorial measures. All the subjects were then grouped into different subtypes on the basis of the highest z-scores obtained for each factor. This procedure gave the opportunity to classify groups of subjects on the basis of the most statistically abnormal substance use cluster. In this way it became possible to resolve the problem of identifying a cut-off for the inclusion of patients in different identified clusters. In this case, to cluster patients in groups, we used correlations between the substances used.

Diagnostic features, specific ADHD symptoms, severity of general psychopathology and patients’ functionality were compared according to the SUD evidenced typology using Student’s T-test for metric and Pearson’s Chi-squared Test for categorial variables.

## 3. Results

### 3.1. Characteristics of the Sample at Treatment Entry

We selected 72 patients with an A-ADHD comorbidity with SUD, of which 52 (72.2%) were males and 20 (27.8%) females (mean age was 27.24; SD = 9.6 years). Among these subjects 22 (31.9 %) were students, 27 (39.1%) were unemployed, 13 (18.8%) were employed, 6 (8.7%) were freelances and 1 (1.4%) was a housewife. Regarding marital status, most of these subjects—61 (87.1%)—were single, 5 (7.1%) were married and 3 (4.3%) divorced. 16 (22.9 %) subjects had legal problems. Regarding psychiatric comorbidities: 10 patients (13.9%) had comorbid Bipolar I Disorder, 26 (36.1%) Bipolar II Disorder, 1(1.4%) Major Depressive Disorder; 25 (34.7%) had comorbid Borderline Personality Disorder, 12 (16.7%) Cyclothymic Disorder and 7 (9.7%) Antisocial Personality Disorder; 20 (27.8%) presented comorbid Generalized Anxiety Disorder, 7 (9.7%) Panic Attack Disorder and 2 (2.8%) subjects had Social Phobia; 13 (18.1 %) had Specific Comorbid Learning Disabilities (DSA), 7 (9.7%) Tic Disorders, and 2 (2.8%) Autism Spectrum Disorder. Three patients (4.2%) had comorbid Anorexia Nervosa, 2 (2.8%) Bulimia Nervosa, and 7 (9.7%) were affected by comorbid Binge Eating Disorder.

### 3.2. Modality of Substance Use

Table 1 shows past and current substance use prevalence and substance use patterns among A-ADHD patients. Regarding substance use, 44 (61.1%) patients used THC, 29 (40.3%) cocaine, 29 (40.3%) MDMA, 21 (29.2%) alcohol, 4 (5.6%) Benzodiazepines (BDZ), 3 (4.2%) amphetamines and 2 (5.6%) opioids both in the past and at treatment entry. As to past but no longer current substance use, 14 (19.4%) patients used THC, 12 (16.7%) cocaine, 12 (16.7%) MDMA, 11(15.3%) amphetamines, 8 (11.1%) alcohol, 8 (11.1%) opioids and 5 (6.9%) BDZ. To complete the picture by adding current but not past use, 5 (6.9%) used THC, 2 (2.8%) cocaine, 2 (2.8%) MDMA, 8 (11.1%) alcohol, 2 (2.8%) BDZ, 1 (1.4%) opioids, while none used amphetamines.

Results of factorial analysis (Table 1) revealed two patterns of substance use: Type 1, prevalent in 44 (61.1%) patients, consisted of Central Nervous System (CNS) stimulants and alcohol in order of value (S-CNS/ALC); and Type 2, prevalent in 28 (38.9%) patients, included THC use and very limited BDZ use. Due to the ordinal nature of the variables, we performed a sensitivity analysis using polychoric correlations for the factor analysis and found no differences with our results (data not shown).

### 3.3. Discriminant Clinical Characteristics according to Substance-Use Clustering

Turning now to demographics, no differences were found between Type 1 and Type 2 substance use regarding gender, marital status or work. Age was higher in Type 2 patients compared with Type 1. Legal problems were significantly higher in the Type 1 group. At multivariate level, two-factor ANOVA analysis (using typology as factor 1, and presence/absence of legal problems as factor 2), did not show any significant group effects (F = 0.33; df = 7; *p* = 0.933), legal problems effects (F = 0.47; df = 7; *p* = 0.849) and group-legal problems effects (F = 0.85; df = 7; *p* = 0.549), regarding all the symptomatological rating scales that were used.

No differences were found in terms of psychiatric comorbidities between the two groups.

Table 2 shows the differences between the Type 1 and Type 2 patients regarding diagnostic assessments. Assessing ADHD diagnosis by applying the DIVA 2.0, no differences were found between the two patterns regarding the ADHD combined, ADHD inattentive and ADHD hyperactive-impulsive subtypes. Evaluating ADHD symptomatology at treatment entry, the two types were similar in terms of inattentive, hyperactive-impulsive and combined symptomatology, as assessed by CAARS-O:S; in addition, the ADHD severity index detected no significant differences between the two patterns of substance use. 

Looking now at psychopathology, no differences were found between the two patterns of use that emerged from the BPRS, BIS-11, HCL-32 and RIPoSt total scores.

In answering the MEQ questionnaire, all patients showed an intermediate chronotype, but Type 1 group scored significantly lower than Type 2 group, so moving towards an evening type. No differences were found between the two patterns of substance use regarding functional impairment as measured by means of the total WHODAS 2.0 score.

## 4. Discussion

In this cross-sectional study we found two distinct patterns of substance use, the first distinguished by the use of both stimulants and alcohol, and the second by the use of THC among A-ADHD patients. Consistently with the literature on ADHD, the substances mostly used by our patients were THC, CNS stimulants and alcohol [20], with less frequent use of BDZs and opioids. To the best of our knowledge this is the first study on A-ADHD patients which has analyzed, by applying factorial analysis, substance use typologies including both past and current use. The concomitant use of stimulants and alcohol is popular and could be due to the specific properties of the substances involved, in so far as psychostimulants are known to produce a rapid addictive feeling of a “high” which can be dampened by the sedative properties of alcohol. Patients belonging to this pattern were, in fact, significantly younger when they attracted our attention by comparison with THC users—a difference that could reasonably be attributed to a higher severity of substance addiction, together with its poly-use consequences. Similarly, legal issues were significantly better represented in the S-CNS/ALC group. Multiple studies [21,22,23,24] showed a high prevalence of ADHD diagnosis in prisoners distinguished by being particularly young when they committed their first crime [25] and showing high rates of recidivism [26]. Previous reports showed that SUDs in ADHD patients, with or without antisocial disorders, seem to increase the risk of violent crimes, especially drug-related ones [27]. The latter could be seen as consequences of the marked emotional dysregulation and impulsivity; affecting subjects with ADHD [28]. Furthermore, substance intoxication and, above all, abstinence could increase the deficits in emotional regulation and therefore increase the risk of aggressive behaviors [29]. Even if substance use in ADHD patients might be seen as a way to reduce manifestations of neurocognitive distress, the combined use and misuse of non-medical prescribed stimulants and alcohol could have the opposite effect, leading to behavioral disturbances, aggressiveness and legal consequences.

In our sample we found that while all patients showed an intermediate sleep chronotype, patients who used mainly THC go to bed earlier. This was expected, as the use of cocaine and/or stimulants, often with subsequent withdrawal, are usually associated with more sleep problems and increased sleep latency [30]. More importantly, the ones who mainly used THC displayed significantly higher total scores, which suggests a probable major role of THC in earlier sleep induction. Current research on cannabinoids use and sleep has given mixed results, with several works supporting the role of the endocannabinoid system in the regulation of circadian rhythm sleep. ADHD patients with poor sleep quality may use cannabinoids for their short-term benefits on sleep, so activating a therapeutic function. However, long-term cannabinoid use may cause addiction to the sleep effects with increased risk of dependence and more sleep disturbances if the drug is stopped. Overall, the global effect seems to be related to the type of cannabinoids, their dosage and route of administration [31].

From a clinical standpoint the most interesting finding was the similar presentation of ADHD symptoms and their severity, independently of substance typologies, at treatment entry. The two patterns were also similar in terms of psychiatric comorbidities, impulsivity emotional dysregulation, general psychopathology, hypomanic symptoms and global functioning. Overall, in the medical literature many authors have studied the influence of ADHD symptomatology on the course of drug addiction in substance abuser samples by mainly focusing on a single substance of abuse, rather than comparisons between different patterns of use. SUDs are often comorbid with a variety of psychiatric disorders, in particular the bipolar ones, in which addiction is associated with more manic switches, a worse psychopathology, less treatment adherence and greater functional impairment [32,33]. A worse course of SUD in ADHD patients compared with other substance users is well established, with an earlier onset of SUD, a more severe symptomatology, higher resistance to therapies and worse drop-out rates [34,35].

Looking at our results we might hypothesize that different SUD typologies do not influence ADHD symptomatology because of the specific effect of substances on ADHD subjects. Even if the results reported in the literature are still controversial as regards the efficacy of stimulants on people with both SUD and ADHD, a recent study from our research group showed a marked reduction of cocaine use concomitant with ADHD improvement in a small sample of A-ADHD patients treated with either atomoxetine or methylphenidate [36]—a finding that suggests the possible benefit of these medications not only in A-ADHD but in SUD components, too. When we talk about stimulants in this specific population, we should take care to correctly differentiate between the two constructs of “relief” and “reward” craving. According to the self-medication theory, a certain number of ADHD patients may choose stimulants driven by a “relief” craving as an attempt to reduce symptoms of inattentiveness and restlessness. On the other hand, other patients who use psychostimulants mostly report a “reward” craving associated with a recreational, pleasurable use. Thus, in ADHD patients, we believe that different substances, in particular stimulants, could be used according to the substance-specific dopaminergic action with autotherapeutic purposes (bringing “self-medication”, to cite Khantzian). In clinical practice we therefore suggest evaluating the type of craving that underlies the use of substances to allow an early diagnosis and a targeted treatment for dual disorder (A-ADHD/SUD) patients. The main result of this study is the evident absence of any symptomatology beyond the two substance use typologies found at treatment entry. One possible explanation for this lack of differences is the following. When entering treatment, the balance of the pre-treatment phase is likely to be lost both for those who “self-medicate” with stimulants, and those who use cannabis for its rewarding effects. The psychopathological decompensation experienced by the patient becomes generalized, and the potential differences can no longer be highlighted. So, it cannot be ruled out that in the pre-treatment phase, these differences do exist. This opens up the possibility that at treatment entry only the differences in MEQ will be noticed, as a result of the chronic use of cocaine and cannabinoids, which give rise to different effects.

We should, in any case, bear in mind that the prolonged use of fast-acting stimulants and polysubstance use in association with the psychopathology specific to ADHD will only lead to more intense dysphoria, more severe mood instability and more deeply negative outcomes.

Polysubstance use, reward and relief craving often coexist in A-ADHD patients but a good knowledge of the dominant typologies of substance use can help clinicians, independently of the quality and severity of symptoms, to better elaborate a treatment strategy and expect different treatment outcomes. If type 1 is dominant, we can anticipate a response to the A-ADHD medications (stimulant drugs), and we should use anti-craving medications for cocaine only if the cessation of cocaine use is still incomplete. Conversely, if type 2 is dominant, we should use anti-craving medications for THC, and, unfortunately, given the scarcity of scientific evidence in this kind of treatment, we shouldn’t expect any important positive pharmacological outcome, especially in dual disorder patients. Our clinical experience usually limits us to combating the psychiatric complications of the THC use. Regarding dominant type 2 A-ADHD patients, there is also the risk of a frequency increase as a result of the new leniency and legal innovations associated with cannabis procurement and use. Nevertheless, cannabis had not been legalized, in Italy, for the time being.

Limitations: Several limitations should be noted in the present study. First, our sample size was small, a datum that limits the generalizability of our results and the power of the study. Indeed, the patients recruited came to our attention as ADHD treatment naïve patients, neither diagnosed nor treated before adulthood. Many patients with ADHD are often misdiagnosed and reached our attention only when the clinical picture was severe and had been complicated by other comorbidities. Consequently, to confirm our hypothesis, more adult ADHD patients with or without a dual disorder need to be enrolled.

Secondly, some of the features assessed, such as impulsiveness, emotional dysregulation and overall functioning, were determined by the answers given to self-report questionnaires, so raising the danger of reporting biases. However, all participants completed the reports under the supervision of a psychiatrist who evaluated them clinically and conducted other valid interviews.

Thirdly, our findings showed a preliminary baseline evaluation of A-ADHD patients at treatment entry, without including a control group. Indeed, this cross-sectional picture is a part of a longitudinal study with multiple follow-up steps that facilitated the evaluation of the trajectories of Substance Use Disorders in A-ADHD patients and helped to make clear how the two disorders are influenced by different psychopharmacological treatments. Moreover, besides SUD typology comparisons, we are also analyzing both similarities and differences in A-ADHD patients with and without a dual disorder at treatment entry.

In conclusion, another significant limitation is the use of a non-random sample. Our hospital is located in Central Italy, which exerts a specific nationwide attraction on patients who have a dual disorder with predominantly psychopathological aspects. Besides, Italian legislation lists centers of excellence for the treatment of adult ADHD in various areas of Italy, and patients typically apply for treatment to the nearest one. So, a bias in favor of the closest officially recommended area cannot be excluded. The risk of a self-selection bias is related to the type of treatment we offer (stimulant medications). So, only patients interested in this kind of medicine may have requested treatment. Finally, our sample did not include patients who choose an “addiction unit” for their treatment; this applied, specially, to those currently using opioids, who, in fact, were only minimally represented in our sample. In Italy, psychiatric units and addiction units work independently, the result being that the level of communication between them is poor. These sample selection biases limit the generalization of our data.

## 5. Conclusions

In our dual disorder (A-ADHD/SUD) patients, those who prevalently use cocaine/alcohol are younger and have more legal issues; patients who only use THC go to bed earlier. Despite these minor differences, our findings show a similar presentation of ADHD symptomatology and severity at treatment entry, independently of substance use typology, so suggesting that differences in the clusters of substances used do not affect the clinical manifestation of A-ADHD at treatment entry.

## Figures and Tables

**Table 1:** Past and current substance use in adults with Attention-Deficit/Hyperactivity Disorder (A-ADHD) with Substance Use Disorders (SUDs).

Substance	Substance Use				Factor Analysis *	
	Never *n* (%)	Past *n* (%)	Current *n* (%)	Past/Current *n* (%)	S-CNS Alcohol	THC BDZ
THC	9 (12.5)	14 (19.4)	5 (6.9)	44 (61.1)	0.08	−0.82
Cocaine	29 (40.3)	12 (16.7)	2 (2.8)	29 (40.3)	0.58	0.07
MDMA	54 (75.0)	12 (16.7)	2 (2.8)	29 (40.3)	0.89	−0.12
Amphetamines	58 (80.6)	11 (15.3)	0 (0.0)	3 (4.2)	0.90	−0.12
Alcohol	35 (48.6)	8 (11.1)	8 (11.1)	21 (29.2)	0.40	0.34
Benzodiazepines	61 (84.7)	5 (6.9)	2 (2.8)	4 (5.6)	0.00	0.79
Opioids	61 (84.7)	8 (11.1)	1 (1.4)	2 (5.6)	0.33	0.26
Variance (%)					31.57	21.57
Patients with prominent use *N* (%)					44 (61.1)	28 (38.9)

*—Extraction Method: Principal Component Analysis. Rotation Method: Varimax with Kaiser Normalization. THC = Cannabinoids; MDMA = 3,4-metilenediossimetanfetamina; S-CNS = Central Nervous System Stimulants; BDZ = Benzodiazepines.

**Table 2:** Discriminant clinical characteristics according to substance use clustering.

Total Score on:	TYPE 1 S-CNS/ Alcohol *N* = 44	TYPE 2 THC BDZ *N* = 28	T	*p*
Demographics	Mean (SD)	Mean (SD)		
Age	25.23 (8.4)	30.39 (10.8)	−2.27	0.026
	*N* (%)	*N* (%)	Chi	*p*
Male gender				
Single civil status	39 (92.9)	22 (78.6)	3.06	0.080
Student/working	24 (58.5)	17 (60.7)	0.03	0.856
Legal problems	14 (31.8)	3 (10.7)	3.90	0.048
Diagnostic	*N* (%)	*N* (%)	Chi	*p*
DIVA 2.0—Combined	35 (81.4)	22 (78.6)	0.08	0.770
DIVA 2.0—Inattentive	5 (11.6)	5 (17.9)	0.54	0.461
DIVA 2.0—Hyper-Impulsive	3 (7.0)	1 (3.6)	0.37	0.543
ADHD symptomatology	Mean (SD)	Mean (SD)	T	*p*
CAARS—Inattentive	17.18 (5.1)	17.39 (5.5)	−0.17	0.868
CAARS—Hyper/Impulsive	15.70 (5.2)	16.11 (7.0)	−0.28	0.781
CAARS—Combined	32.48 (9.0)	33.39 (11.3)	−0.38	0.704
CAARS Index	22.66 (5.2)	24.18 (5.5)	−1.17	0.244
RiPoSt-40	219.14 (43.8)	224.43 (47.3)	−0.48	0.634
BIS-11	83.33 (10.3)	82.89 (12.5)	0.16	0.874
General Psychopathology	Mean (SD)	Mean (SD)	T	*p*
BPRS	41.98 (16.6)	43.68 (15.1)	−0.44	0.662
HCL-32	18.86 (5.9)	16.61 (5.9)	1.57	0.120
MEQ	42.47 (13.0)	50.11 (7.3)	−3.07	0.003
Functionality	Mean (SD)	Mean (SD)	T	*p*
WHODAS 2.0	2.59 (0.64	2.69 (0.90	−0.56	0.577

THC = Cannabinoids; BDZ = Benzodiazepines; DIVA 2.0 = Diagnostic Interview for ADHD in adults; ADHD = Attention Deficit/Hyperactivity Disorder; CAARS-O:S = Conner’s Adult ADHD Rating Scales–Observer; BIS-11 = Barratt Impulsiveness Scale; BPRS = Brief Psychiatric rating scale; RIPoSt-40 = Reactivity Intensity Polarity Stability Questionnaire; (WHODAS = World Health Organization Disability Assessment Schedule; HCL-32 = Hypomania Checklist; MEQ = Morningness-Eveningness Questionnaire.
